# Mechanisms of NK Cell Activation and Clinical Activity of the Therapeutic SLAMF7 Antibody, Elotuzumab in Multiple Myeloma

**DOI:** 10.3389/fimmu.2018.02551

**Published:** 2018-11-05

**Authors:** Kerry S. Campbell, Adam D. Cohen, Tatiana Pazina

**Affiliations:** ^1^Blood Cell Development and Function Program, Fox Chase Cancer Center, Philadelphia, PA, United States; ^2^Abramson Cancer Center, University of Pennsylvania, Philadelphia, PA, United States; ^3^FSBSI “Institute of Experimental Medicine”, St. Petersburg, Russia

**Keywords:** multiple myeloma, SLAMF7, elotuzumab, NK cells, ADCC, ADCP, macrophage

## Abstract

Multiple myeloma (MM) is a bone marrow plasma cell neoplasm and is the second most-common hematologic malignancy. Despite advances in therapy, MM remains largely incurable. Elotuzumab is a humanized IgG1 monoclonal antibody targeting SLAMF7, which is highly expressed on myeloma cells, and the antibody is approved for the treatment of relapsed and/or refractory (RR) MM in combination with lenalidomide and dexamethasone. Elotuzumab can stimulate robust antibody-dependent cellular cytotoxicity (ADCC) through engaging with FcγRIIIA (CD16) on NK cells and antibody-dependent cellular phagocytosis (ADCP) by macrophages. Interestingly, SLAMF7 is also expressed on cytolytic NK cells, which also express the requisite adaptor protein, EAT-2, to mediate activation signaling. Accumulating evidence indicates that antibody crosslinking of SLAMF7 on human and mouse NK cells can stimulate EAT-2-dependent activation of PLCγ, ERK, and intracellular calcium mobilization. The binding of SLAMF7 by elotuzumab can directly induce signal transduction in human NK cells, including co-stimulation of the calcium signaling triggered through other surface receptors, such as NKp46 and NKG2D. In RRMM patients, elotuzumab monotherapy did not produce objective responses, but did enhance the activity of approved standard of care therapies, including lenalidomide or bortezomib, which are known to enhance anti-tumor responses by NK cells. Taken together, these preclinical results and accumulating experience in the clinic provide compelling evidence that the mechanism of action of elotuzumab in MM patients involves the activation of NK cells through both CD16-mediated ADCC and direct co-stimulation via engagement with SLAMF7, as well as promoting ADCP by macrophages. We review the current understanding of how elotuzumab utilizes multiple mechanisms to facilitate immune-mediated attack of myeloma cells, as well as outline goals for future research.

## Introduction

Multiple myeloma (MM) is a deadly hematopoietic cancer characterized by the expansion of monotypic plasma cells in the bone marrow, accumulation of monoclonal immunoglobulin in the serum, and end-organ damage such as anemia, lytic bone lesions, and renal dysfunction (1). It is estimated that almost 31,000 cases of MM will be diagnosed in the U.S. in 2018 and almost 13,000 will die of the disease. Incidence increases with age, which accounts for a steadily rising prevalence of MM overall (2). Rates of median survival are improving, with overall 5-year survival of about 50%, although survival is better in younger patients (2). Nonetheless, MM is still a largely incurable disease, highlighting the need for improved therapeutic options, which may include new agents with novel mechanisms of action and innovative combination therapies.

A variety of recently-developed therapies have contributed to the extended survival of MM patients, including proteasome inhibitors (bortezomib, carfilzomib, and ixazomib), immunomodulatory imide drugs (IMiDs; namely thalidomide, lenalidomide and pomalidomide), and monoclonal antibodies (daratumumab and elotuzumab). Clinical results with these therapies have been previously summarized in a variety of quality reviews (3–6) and will not be further discussed here. Importantly, however, optimal long-term control of MM requires combinations of two or even three different classes of drugs (7). Furthermore, in contrast to older MM therapies such as steroids or cytotoxic chemotherapies, these newer therapies can mediate their anti-myeloma activity not just by acting directly on the myeloma cell, but also through modulation of the patient's immune system (8). Thus, gaining a greater understanding of the mechanisms of action of these new therapies, and particularly how they impact host innate and adaptive immunity, will be critical to further developing optimal combinations for treatment.

Here, we will review current understanding of the mechanisms by which elotuzumab promotes immune responses toward MM, especially through facilitating NK cell-mediated anti-tumor activity. We further summarize clinical results from the use of elotuzumab in combination immunotherapies and discuss how the immune potentiating mechanisms may be contributing to anti-tumor responses in patients. While the CD38 targeting antibody, daratumumab, shares some mechanistic attributes with elotuzumab, we will only touch upon some aspects of the effects of daratumumab, in view of recently published reviews on the topic (9, 10).

## NK cells and multiple myeloma

NK cells are believed to play important roles in immune surveillance of cancer, limiting neoplastic progression, and effectors of anti-tumor therapies (11, 12). Their stimulation is triggered upon recognition of certain ligands on tumor cells by cell surface activating receptors [including NKG2D, CD16, 2B4, NKp80, and DNAM-1, and natural cytotoxicity receptors (NCR: NKp30, NKp44, and NKp46)] (13). NK cell stimulation is, however, tightly regulated by their expression of major histocompatibility class I (MHC-I)-binding inhibitory receptors [killer cell Ig-like receptors (KIR; CD158), CD94/NKG2A heterodimers, and ILT2/LIR1/CD85j], which efficiently block NK cell activation toward MHC-I-expressing normal cells (12). Therefore, when an NK cell conjugates with an abnormal tumor cell lacking MHC-I and expressing ligands for activating receptors, the inhibitory receptors are not engaged, and unsuppressed activating signals trigger targeted attack of the conjugated cell.

The importance of NK cells in mediating anti-myeloma activity has been demonstrated in several ways. A graft-vs.-myeloma effect has been shown by the differences in post-allogeneic stem cell transplant relapse rates based on the inherited repertoire of *KIR* genes expressed by donor NK cells (14, 15), indicating a role for NK cell-mediated suppression of relapse. NK cells can clearly mediate direct cytotoxicity and ADCC against myeloma cells *in vitro* and *in vivo* (16–19). This response depends on the expression of activating receptors, such as NKG2D, DNAM-1, and the NCRs, on the NK cells, along with their respective ligands on the myeloma cells (16, 17, 20). Several studies have now shown that the balance of activating and inhibitory NK cell receptors and ligands is significantly altered in MM patients, especially in advanced disease (16, 21–26). For example, myeloma cells derived from a patient late in disease course (from a pleural effusion) expressed much higher levels of MHC-I (an inhibitory ligand) and lower levels of MICA (a ligand for the NK cell activating receptor, NKG2D) and were much more resistant to NK cell-mediated lysis than myeloma cells derived earlier from the bone marrow of the same patient (16). In addition, MICA can be shed off the myeloma cell surface and reportedly down-regulate or block engagement of the activating NKG2D receptor on NK and T cells (27, 28). This mutual “immuno-editing” of receptor and ligand expression on the surface of NK and myeloma cells, respectively, implies a strong selective pressure of NK cells on the tumor, and suggests that strategies augmenting NK cell activity may overcome this immune evasion and eliminate MM. Finally, data that currently-used therapies (e.g., melphalan, bortezomib, lenalidomide) can augment NK cell-mediated cytotoxicity against MM (3, 20, 24, 26, 29–34) provide strong support for exploring combinations of NK cell-targeted therapies with these active anti-myeloma agents.

## SLAMF7 as a prominent biomarker and potential therapeutic target on myeloma cells

Signaling Lymphocyte Activation Marker Family member 7 (SLAMF7) was found highly expressed on human plasma cells and corresponding myeloma cells (18, 19). While the physiological function of SLAMF7 on plasma cells is still unknown, the high expression on myeloma cells raised interest as a therapeutic antibody target. Hsi and colleagues detected high levels of SLAMF7 mRNA in CD138^+^ plasma cells from healthy donors, patients with MGUS, smoldering myeloma and newly diagnosed patients, whereas NK cells expressed a substantially lower level of SLAMF7 mRNA (18). High expression on myeloma cells was also found in MM patients, regardless of cytogenetic abnormalities. Examination of SLAMF7 protein expression on MM, other plasma cell tumors, and normal tissues was consistent with mRNA expression patterns, where strong surface staining was found on plasmacytomas (18), most myeloma cells from bone marrow biopsies, neoplastic plasma cells from most lymphoplasmacytic lymphoma, and some peripheral T cell lymphomas. Importantly, SLAMF7 expression was preserved on myeloma cells at significant levels upon relapse in most patients (18). Tai et al. further confirmed that SLAMF7 mRNA is expressed in CD138^+^ tumor cells from more than 97% of MM patient analyzed and surface SLAMF7 protein was detected on several myeloma cell lines and 12 representative MM tumor samples (19). The same study also detected soluble SLAMF7 in 32 of 54 serum samples from MM patients, but not healthy donors, which they suggest could serve as a biomarker of active disease (19). It was also shown that myeloma cells with t(4;14) translocations (found in about 15% of MM patients) express higher levels of SLAMF7 mRNA and surface protein, which appears to be due to overexpression of MMSET (35). Interestingly, shRNA-mediated knockdown of SLAMF7 expression in t(4;14) myeloma cells reduced colony formation and induced G1 arrest and apoptosis, indicating that maintaining high SLAMF7 expression promotes growth of these myeloma cells (35). A recent analysis of gene expression data in hematopoietic malignancies confirmed high SLAMF7 expression on myeloma tumors, but also identified high SLAMF7 expression on tumors in patients with myelodysplastic syndrome, chronic lymphocytic leukemia, and diffuse large B cell lymphoma (36). This result suggests that SLAMF7 may also be a useful diagnostic marker and therapeutic target in other hematopoietic cancers. However, the biological role of SLAMF7 on the pathogenesis of these tumors types has not been thoroughly evaluated.

## Elotuzumab as a new therapeutic to target multiple myeloma

### Preclinical studies: ADCC by NK cells as a major mechanism of action

Elotuzumab (Elo; formerly HuLuc63) is a humanized IgG1 monoclonal antibody that was developed to target SLAMF7. HuLuc63 was originally engineered by PDL BioPharma as a humanized version of the SLAMF7 monoclonal antibody, MuLuc63, which was originally generated in BALB/c mice (18, 19). HuLuc63 binds to the carboxy-terminal Ig-like constant 2 (C2) domain of SLAMF7, which encompasses amino acids 170-227 (U.S. patent 7842293B2). It is important to note that HuLuc63 does not cross-react with other SLAM family proteins and did not activate complement-dependent lysis or direct cytotoxicity of myeloma cells (19, 37).

From the earliest studies, Elo was found to promote antibody-dependent cellular cytotoxicity (ADCC) of myeloma cells by NK cells both *in vitro* and *in vivo* (18, 19, 38). Hsi et al. found that HuLuc63 induced specific myeloma cell lysis in multiple assays using PBMCs or purified NK cells from healthy allogeneic donors or autologous NK cells toward myeloma cells or myeloma cell lines (18). HuLuc63 was also shown to induce similar lysis of patient myeloma cells by NK cells from allogeneic healthy donors as compared to NK cells from the same MM patient, even in patients who were resistant to conventional therapy (18). Furthermore, HuLuc63 was significantly more effective in inducing ADCC responses by NK cells than another chimerized SLAMF7 antibody (human IgG1-human Fc/mouse variable regions) named ChLuc90 (18, 19, 39). Tai et al. also demonstrated that HuLuc63 stimulated ADCC responses by NK cells from healthy donors to a variety of myeloma cell lines, and they further showed strong ADCC of autologous myeloma cells by NK cells from MM patients, even if patients were resistant to conventional therapies (19).

Preclinical *in vitro* studies also found minimal loss of NK cells in PBMC treated with Elo, indicating that the antibody does not induce significant NK cell fratricide upon binding to SLAMF7 on NK cells themselves. Treatment of whole blood overnight with 100 or 200 μg/ml HuLuc63 resulted in a loss of only 20% of NK cells (18). Another study found that in cultures of PBMC overnight with up to 100 μg/ml Elo, NK cell viability was retained at >95% (39). Fratricide was also not observed when purified NK cells were exposed to 100 μg/ml Elo for 72 h, possibly due to upregulation of MHC class I on the NK cell surface as a ligand for inhibitory signaling (37). Similarly, Elo therapy results in only a transient loss of NK cells in peripheral blood of patients within hours after the initial dose that recovers over time (40). A parallel loss of T and B cells also occurred, which the authors attributed to an early increase in serum levels of the chemokine IP-10 (CXCL10), which induces migration of lymphocytes and myeloid cells. A similar transient early loss and recovery of NK cells in peripheral blood was also noted in another clinical trial (41). Thus, it appears that NK cell fratricide is minimal in patients treated with Elo. In contrast, patients treated with daratumumab exhibit significant loss of NK cells in peripheral blood, due to ADCC-mediated fratricide (42), although daratumumab-treated patients alternatively benefit from depletion of immunosuppressive regulatory T cells, regulatory B cells, and myeloid-derived suppressor cells to boost anti-myeloma immune responses (9).

### Preclinical studies of combination therapies with elotuzumab

The early study by Tai et al. showed enhanced NK cell-mediated ADCC responses by HuLuc63 if the myeloma cell lines were pretreated with several drugs, including bortezomib, dexamethasone, and lenalidomide (19). These experiments provided the first preclinical evidence for the use of Elo in combination with other therapies to treat MM patients.

Van Rhee at al. subsequently tested the effect of Elo in combination with the 26S proteasome inhibitor, bortezomib, in a severe combined immunodeficiency (SCID)-human mouse xenograft model engrafted with primary myeloma cells (38). Treatment of mice with MuLuc63 (the parental mouse mAb from which Elo was derived) alone promoted significant reductions of tumor volume and human IgG in serum, with equivalent responses to high- or low-risk myeloma samples (38). Treating a MM target cell line or autologous myeloma cells with bortezomib was found to enhance *in vitro* susceptibility to Elo-mediated ADCC by NK cells (38). This increased susceptibility is consistent with reports that bortezomib treatment reduces expression of the NK cell inhibitory receptor ligand, MHC class I, and increases expression of ligands for the activating receptor, NKG2D (3, 30, 43). Van Rhee et al. also showed that mice treated with the combination of Elo plus bortezomib had significantly more efficient anti-tumor response in an OPM2 myeloma cell line xenograft mouse model, compared to treatment with either agent alone (38). The group additionally showed that SLAMF7 expression on myeloma cells was not affected in patients treated with bortezomib (38).

Balasa et al. investigated *in vitro* and *in vivo* effect of Elo in combination with lenalidomide on NK cell activation, cytokine production and myeloma cell death (44). Lenalidomide is a member of the IMiD family, which also includes thalidomide and pomalidomide, that can augment function of T and NK cells, suppress angiogenesis, and directly restrain myeloma cell growth (3). Treatment of the OPM2 xenograft mouse model with Elo plus lenalidomide resulted in significantly greater reduction in tumor volume and increased infiltration of NK cells into the tumor microenvironment (44). The combination of Elo with lenalidomide in co-cultures of peripheral blood mononuclear cells (PBMC) and myeloma cells also increased upregulation of NK cell activation marker, adhesion molecules, and cytokine production (IFN-γ, TNF-α, IL-2), as compared to either agent alone (44). These effects required the Fc domain of Elo, indicating the primary role of NK cell-mediated ADCC, and were enhanced by IL-2 production by CD56^+^ T cells within the PBMC. In addition TNF-α production contributed significantly to NK cell activation and myeloma cell cytotoxicity (44). Therefore, Elo in combination with lenalidomide was highly effective and appeared to primarily benefit from NK cell activation in response to IL-2 produced by T cells and TNF-α production by monocytes and NK cells.

Bezman et al. studied the *in vivo* impact of a murine IgG2a-modified version of Elo (Elo-g2a) in xenograft mouse models using immunocompetent mice and syngeneic mouse tumors expressing human SLAMF7 (25). Treatment with Elo-g2a significantly reduced tumor volume and the effect was reversed if NK cells were depleted from the mice or mice were instead treated with a Fc mutant form of Elo-g2a that cannot bind Fcγ receptors (25). In these xenograft mouse models, PD-1 expression was found to be increased on tumor-infiltrating T cells and the tumors expressed PD-L1. Consistent with this observation, the combined treatment with Elo-g2a and PD-1 antibody resulted in significantly reduced tumor volume and increased survival compared to either agent alone (25). The combination therapy resulted in increased expression of IFN-γ, TNF-α, CD69, and CD107a degranulation marker on tumor-infiltrating NK cells, as compared to treatment with either antibody alone. Furthermore, long-term surviving mice from one of these mouse models were protected from subsequent challenge with the same tumor, indicating that immunological memory had been established in response to the combination therapy (25). The results suggest that the combination of Elo with PD-1/PD-L1 blocking antibody therapy may also be an effective strategy in treating MM patients.

Taken together, these combination therapy preclinical studies provided further evidence that NK cells play an important role in the mechanism by which Elo effectively reduces MM tumor burden in mice. In particular, both bortezomib and lenalidomide are known to boost NK cell function, which could contribute to better ADCC responsiveness (3). The potentiation of Elo effectiveness by PD-1 blockade also corresponded to enhanced NK cell responsiveness, likely due to overriding this important immune checkpoint on T cells and perhaps NK cells (23, 45, 46).

### Clinical trials with elotuzumab to treat multiple myeloma (summarized in Table 1)

Zonder et al. performed the first-in-human phase I study to evaluate safety, tolerability, pharmacokinetics and pharmacodynamics of Elo (40). Thirty-five relapsed/refractory (RR) MM patients were treated with 0.5–20 mg/kg of Elo every 2 weeks. Elo was generally well-tolerated, even at the highest dose, and saturation of over 95% of SLAMF7 receptors on bone marrow plasma cells was achieved at 10 and 20 mg/kg (40). However, no objective anti-myeloma response was observed in the MM patients treated with Elo as a single agent in this highly pre-treated population, despite reaching high SLAMF7 saturation with Elo (40).

**Table 1:** Comparisons of response rates (RR) progression free survival (PFS) in elotuzumab clinical trials.

**Phase**	Regimen^*^	N^*^	**Overall RR**	**Median PFS**	**References**
I	Elo	35	0%	N/A	(40)
I	Elo/Bor	27	48%	9.46 months	(41)
II	Elo/Bor/Dex vs. Bor/Dex	150	66 vs. 63%	9.7 vs. 6.9 months	(47)
I	Elo/Len/Dex	28	82%	N/A	(48)
Ib/II	Elo/Len/Dex	73	84%	28.6 months	(49)
III	Elo/Len/Dex vs. Len/Dex	646	79 vs. 66%	19.4 vs. 14.9 months	(50)
II	Elo/Td/Dex	40	38%	3.9 months	(51)
II	Elo/Pom/Dex vs. Pom/Dex	117	53 vs. 26%	10.3 vs. 4.7 months	(52)

* *Elo, elotuzumab; Bor, bortezomib; Dex, dexamethasone; Len, lenalidomide; Td, thalidomide; Pom, pomalidomide; n, number of evaluable patients*.

Jakubowiak et al. performed the first phase I trial of Elo in combination with bortezomib in RRMM (41). The combination was safe and showed promising activity with an objective response in 48% of 27 patients, including partial responses in patients refractory to previous bortezomib therapy. Serum concentrations of Elo in these patients were >100 ug/ml at doses of 10 or 20 mg/kg with 80 or 95% median saturation of SLAMF7 on CD38^+^ myeloma cells in bone marrow, respectively (41). The most frequent adverse events were lymphopenia and fatigue.

A subsequent phase II study comparing the effects of Elo plus bortezomib/dexamethasone (Bor/Dex; 75 patients) vs. Bor/Dex alone (75 patients) for RRMM was also reported by Jakubowiak et al. (47). At 1 year, progression free survival rate was 39% for the Elo-treated group compared to 33% for Bor/Dex alone, while the rate was 18 vs. 11%, respectively at 2 years (47). Strikingly, patients homozygous for the high affinity polymorphic variant of FcγRIIIa (CD16 158V/V) in the Elo/Bor/Dex group had a median progression free survival of 22.3 months compared to only 9.8 months for patients homozygous for the lower affinity variant (CD16 158 F/F) (47). This result suggests an important role for CD16 in the mechanism of Elo activity in MM patients, analogous to enhanced ADCC and clinical response to rituximab in follicular lymphoma patients having CD16 158/V/V genotype (53, 54). No significant increase in toxicity was noted when Elo was added to Bor/Dex therapy, with the most common side effects being infection, diarrhea, and thrombocytopenia (47).

The initial phase I trial of the combination of Elo/Lenalidomide/Dexamethasone (Elo/Len/Dex) was carried out by Lonial et al. (48). In that study, 29 previously-treated advanced MM patients were treated with 5, 10, and 20 mg/kg of Elo. Neutropenia and thrombocytopenia were the most frequent adverse events. Outcomes were encouraging, with 82% of patients achieving an objective response, including patients who had received prior thalidomide, bortezomib or lenalidomide therapy (48). Treatment with 10 or 20 mg/ml Elo resulted in full saturation of more than 80% of SLAMF7 binding sites on CD38^+^ myeloma cells and consistently achieved Elo serum concentration of more than 70 ug/ml in patients, with peak concentrations of up to 1 mg/ml in serum (48).

Richardson et al. subsequently reported on the phase II portion of this trial (49). In this phase, 73 patients were randomized to either 10 or 20 mg/kg of Elo, in combination with Len/Dex. Objective responses were observed in 84% of patients with a better response in the low-dose group, and no dose-limiting toxicities observed. Median progression free survival was 29 months (10 mg/kg, 32 months; 20 mg/kg, 25 months) (49). Retrospective analysis of bone marrow samples obtained from this trial revealed increased infiltration of CD56^dim^CD16^+^ NK cells exhibiting higher expression of the adhesion molecule, CD54 (ICAM-1), and concomitant reduction in CD45^dim^CD138^+^ myeloma cells at cycle 1 day 22, as compared to baseline (25).

Lonial et al. performed a phase III Elo/Len/Dex trial (ELOQUENT-2) comparing treatment of 321 RRMM patients with 10 mg/kg Elo/Len/Dex and a control arm of 325 patients treated with Len/Dex alone (50). At 1 year, progression free survival in the Elo-treated patients was 68% compared to 57% in the Len/Dex control group, and 41 and 27%, respectively at 2 years. Follow up analysis at 3 years found 26% progression free survival for the Elo-treated patients vs. 18% for the control group (55). Overall response rate was 79% for the Elo-treated patients vs. 66% in the control group (50). Common adverse events were lymphocytopenia, anemia, thrombocytopenia, neutropenia, fatigue and diarrhea, but there was no evidence of autoimmunity in Elo-treated patients. The significant reduction in disease progression and death in the Lonial et al. study (50) was instrumental in subsequent U.S. FDA approval in November 2015 for the use of the Elo/Len/Dex combination to treat RRMM patients that have received one to three previous lines of therapy.

In addition to the combined use of Elo with Lenalidomide and dexamethasone, clinical trials are underway combining Elo with related IMiD drugs, thalidomide and pomalidomide. The combination of Elo with thalidomide/dexamethasone was shown to be safe and effective in a phase II study by Mateos et al. (51). Furthermore, results from the randomized phase II ELOQUENT-3 trial comparing Elo/pomalidomide/dexamethasone with pomalidomide/dexamethasone in RRMM patients were recently presented at the 2018 European Hematology Association meeting by Dimopoulos et al. (52). Overall response rate of the Elo-treated group was 53% compared to 26% for the control group, and median progression free survival was 10.3 vs. 4.7 months, respectively. Therefore, combination therapies of Elo, particularly with IMiDs have demonstrated significant clinical activity in RRMM disease and additional combination studies, including in newly-diagnosed patients, are underway.

## SLAMF7 expression and signaling in leukocytes (summarized in Table 2)

### Expression and structure of SLAMF7

SLAMF7 (CD319) was originally discovered as a CD2-related receptor by the labs of Marco Colonna, who called it CRACC (CD2-like receptor activating cytotoxic cells) (57), and Porunelloor Mathew, who named it CS1 (CD2 subset 1) (56). The gene encoding SLAMF7 is found on chromosome 1 in humans in a locus at 1q23-24 that contains most of the other SLAM (signaling lymphocyte activation molecule) family receptors (56, 57, 70). In addition to plasma cells and myeloma cells, SLAMF7 is expressed in healthy donors on essentially all CD56^dim^ NK cells, the majority of CD56^bright^ NK cells, many CD56^+^ T cells, mature dendritic cells, and small subsets of CD4^+^ T cells and B cells (18, 39, 57). IL-12 produced by dendritic cells has been shown to increase expression of SLAMF7 on NK cells (71). B cells have also been reported to increase SLAMF7 expression upon activation with various stimuli (57, 70, 72). While early studies did not detect SLAMF7 on CD14^+^ monocytes (18, 57), a more recent report found significant expression on most non-classical (CD14^low^CD16^+^) and a fraction of intermediate (CD14^+^CD16^+^) monocytes (39). In addition, SLAMF7 is preferentially expressed at higher levels on M1 macrophages, as compared to M2 macrophages in humans (73).

**Table 2:** Main biological functions of SLAMF7.

**Function**	**References**
SLAMF7 is a self-ligand	(56–58)
SLAMF7-S isoform lacking ITSM-like sequence	(59)
NK cells activation upon SLAMF7 engagement with mAbs	(57–62)
SLAMF7 preferentially recruits EAT-2 over SAP	(57, 61–65)
EAT-2 stimulates calcium mobilization and ERK activation	(63, 66)
SLAMF7 mediates inhibitory function in the absence of EAT-2	(37, 61, 67)
Memory-like NK cells lack EAT-2	(68, 69)
SLAMF7 signals through association with Mac-1 in macrophages	(36)

Mature SLAMF7 is expressed as a 66 kDa glycoprotein (57). Similar to most SLAM family members, the extracellular domain contains an amino terminal Ig-like variable (V) domain and a carboxy-terminal Ig-like constant 2 (C2) domain and can interact with other SLAMF7 extracellular domains via the V domains as self-ligands (56, 57, 58). The cytoplasmic domain of SLAMF7 contains four tyrosine residues, one of which (TVY_304_STV) is within an immunoreceptor tyrosine-based switch motifs (ITSM; T-V/I-Y-x-x-V/I) and another that is embedded in a similar ITSM-like sequence (TEY_284_DTI). ITSM sequences are also found in most SLAM family receptors, and have capacity to switch between activating and inhibitory signaling (64). Tyrosine phosphorylation is mediated by Src family kinases, including Fyn, Lyn, and Src (67, 74). Upon tyrosine phosphorylation, an ITSM can recruit either SLAM adaptor protein (SAP, encoded by *SH2D1A*) or EWS-FLI1 activated transcript 2 (EAT-2, encoded by *SH2D1B*) (65) to mediate activation signaling in human leukocytes (mice also express ERT, which is not found in humans). Interestingly, in addition to this ITSM-containing form of SLAMF7 with a long cytoplasmic domain (SLAMF7-L), a mRNA splice variant encodes a receptor with a shorter cytoplasmic domain (SLAMF7-S) lacking the two ITSM-like sequences and possessing an alternative ITSM-like motif (SKYGLL) (59). NK cells predominantly express the SLAMF7-L variant, which exclusively exhibits activation signaling properties (59). It is currently unclear if a subset of NK cells can predominantly express SLAMF7-S or if some individuals preferentially express this truncated isoform.

### SLAMF7 signaling and the importance of EAT-2

From early studies, SLAMF7 was found to activate NK cells upon engagement with monoclonal antibodies (mAb), SLAMF7-Ig fusion protein, or exposure to SLAMF7^+^ target cells (57, 58, 60, 61). Crosslinking SLAMF7 with mAb induced the serine phosphorylation of Akt and ERK and tyrosine phosphorylation of phospholipase C (PLC)-γ1, PLC-γ2, c-Cbl, Vav1, and SHIP-1 (57, 62). Engagement of SLAMF7 with biotinylated antibody + streptavidin crosslinking was shown to stimulate intracellular calcium mobilization in mouse NK cells that required EAT-2 (61), and engaging human SLAMF7 in a rat NK cell line with the 1G10 mAb + secondary crosslinking antibody induced a strong calcium signal (59). In contrast, Pazina did not observe any elevation of intracellular calcium when primary NK cells from healthy donors were treated with biotinylated Elo ± streptavidin (39), indicating that calcium signaling through engaging SLAMF7 does not occur in normal human NK cells or Elo binds to an epitope on SLAMF7 that is incapable of properly engaging the receptor to directly mediate calcium signaling.

Tyrosine phosphorylated SLAMF7 was found to preferentially recruit EAT-2, but not SAP, at tyrosine(Y)-304 within the ITSM (61, 62). Although SAP association with SLAMF7 has also been reported (59, 62), EAT-2 is recruited to tyrosine phosphorylated SLAMF7 at >100-fold higher affinity than SAP (63). In addition, antibody engagement of SLAMF7 stimulated comparable levels of cytotoxicity by NK cells from both SAP-deficient X-linked lymphoproliferative disease patients and healthy donors, further indicating that SLAMF7 activation signaling is independent of SAP (57). EAT-2 expression was shown to promote the tyrosine phosphorylation of SLAMF7 by Src family kinases in one study (62), but this was not confirmed in another study (61). Importantly, EAT-2 is strongly expressed in NK cells, somewhat in γδ T cells, but not in B, CD4^+^ T, CD8^+^ T, or dendritic cells (62, 75). Interestingly, CD16^+^ non-classical monocytes express both SLAMF7 and EAT-2 (39, 76), indicating that SLAMF7 is likely signaling competent in this population of monocytes that is also capable of mediating antibody-dependent cellular phagocytosis (ADCP) through CD16 (77).

**Figure 1:**
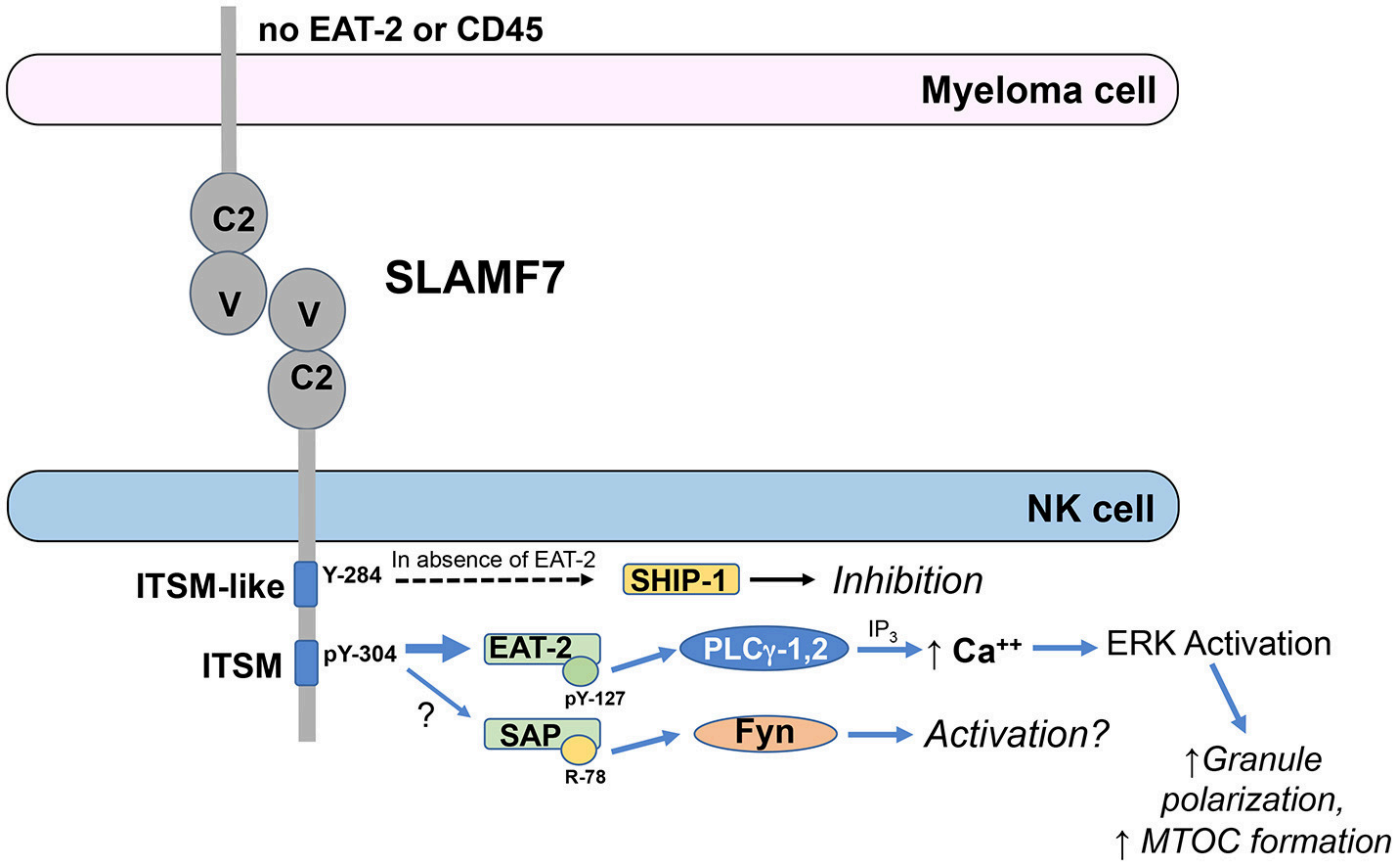
ITSM-mediated signaling by SLAMF7 in NK cells. EAT-2 is predominantly recruited to phosphorylated tyrosine (pY)-304 within the ITSM of SLAMF7 (TVYSTV; numbering follows NCBI reference sequence NP_067004.3 and UniProt Q9NQ25-1) and PLCγ-1 and PLCγ-2 are recruited to pY-127 on EAT-2. Activated PLCγ generates inositol trisphosphate (IP_3_), which induces release of calcium from the endoplasmic reticulum into the cytosol, causing activation of ERK and downstream co-stimulation of polarization of the microtubular organizing center and cytolytic granules toward the tumor cell to enhance cytotoxicity. SAP has also been reportedly recruited to pY-304 at lower affinity and can recruit Fyn to arginine (R)-78, resulting in downstream activation, although the functional relevance of SAP recruitment is unclear. SHIP-1 can also be recruited to SLAMF7 to mediate inhibitory signaling in cells lacking EAT-2 expression and this recruitment requires Y-284 in the ITSM-like sequence on SLAMF7, but direct binding has not been demonstrated to that site to date. These signaling pathways are abrogated in myeloma cells, due to their lack of EAT-2 and CD45 expression.

EAT-2 is a small cytosolic adaptor protein that consists of an SH2 domain, which binds to Y304 on SLAMF7, and a short C-terminal sequence including a tyrosine at position 127 (Y127), which is required for SLAMF7 activating function (61). When EAT-2 is tyrosine phosphorylated on Y127, it can subsequently recruit PLC-γ1 and PLC-γ2 to stimulate downstream intracellular calcium mobilization and ERK activation (63, 66). Consistent with these findings, expression of EAT-2 in the EAT-2-deficient human NK cell line, YT-S, enhanced tyrosine phosphorylation of PLC-γ1 and Cbl and serine phosphorylation of ERK in response to crosslinking the SLAM family receptor, 2B4 (66). Functionally, EAT-2 signaling in NK cells can enhance polarization of cytolytic granules and the microtubular organizing center (MTOC) toward target cells and degranulation responses, but does not promote conjugate formation with target cells (66). The known SLAMF7 signaling mechanisms are outlined in Figure 1.

SLAMF7 has also been observed to exhibit some inhibitory function in NK cells from EAT-2-deficient mice or the human NK cell line, YT-S, in the absence of EAT-2 (61). Therefore, available data indicate that EAT-2 is required for activating function of SLAMF7, but the receptor can mediate inhibitory function in the absence of EAT-2. Early work was unable to demonstrate co-immunoprecipitation of the inhibitory phosphatases, SH2 domain-containing 5′-inositol phosphatase (SHIP)-1, SH2 domain-containing tyrosine phosphatase (SHP)-1, or SHP-2 with tyrosine phosphorylated SLAMF7 in the EAT-2-expressing human NK cell line, NK-92, treated with pervanadate (57, 59), but a subsequent study by Guo et al. demonstrated recruitment of SHIP-1 to mouse SLAMF7 when expressed in the EAT-2-deficient human NK cell line, YT-S, and engaged with a SLAMF7 mAb (67). The stimulation of these YT-S cells with SLAMF7 mAb also induced tyrosine phosphorylation of SHIP-1, but this did not occur if Y261 (analogous to human Y284) on the mouse SLAMF7 was mutated to phenylalanine, even though direct binding to Y261 was not demonstrated (67). There is also no evidence that EAT-2 and SHIP-1 compete for binding to SLAMF7. These results indicate that the inhibitory function of mouse SLAMF7 in the absence of EAT-2 is mediated by SHIP-1 and requires Y261 (human Y284) on SLAMF7. Interestingly, “adaptive” or “memory-like” NK cells have been reported to lack expression of EAT-2 (68, 69). It is currently unclear whether the adaptive/memory-like cells, which exist as a subset of the NK cell repertoire in many human cytomegalovirus seropositive individuals, are inhibited toward SLAMF7^+^ target cells or have unique responsiveness to Elo compared to conventional NK cells.

Importantly, plasma cells and myeloma cells express high levels of SLAMF7, but lack expression of EAT-2, thereby compromising their activation signaling capacity through SLAMF7 (37, 67). This lack of EAT-2 suggested that SLAMF7 may function as an inhibitory receptor in myeloma cells. However, while treatment with SLAMF7 antibody (162) and secondary crosslinker can induce varying levels of tyrosine phosphorylation of the receptor in different myeloma cell lines, SHIP-1 was only minimally tyrosine phosphorylated, if at all (67). The defective tyrosine phosphorylation of SHIP-1 was attributed to lack of CD45 expression in myeloma cells, since CD45 is a tyrosine phosphatase required for maintaining activity of Src family kinases that mediate the tyrosine phosphorylation of SHIP-1 (67). Thus, SLAMF7 does not have activation or inhibitory signaling function in myeloma cells, due to the lack of EAT-2 to mediate activation and the lack of CD45 to maintain Src family kinases in an active state that is required to phosphorylate SLAMF7 and the inhibitory SHIP-1 phosphatase. Consistent with these findings, Elo was found to be incapable of inducing proliferation or apoptosis of myeloma cell lines, even in plate bound form (25, 67).

Recent intriguing work by Chen et al. established a novel mechanism for SLAMF7 signaling through physical association with the integrin receptor CD11b/CD18 (Mac-1) in the plasma membrane on the surface of macrophages (36). This group found that SLAMF7 serves as a key receptor promoting the phagocytosis of SLAMF7-expressing hematopoietic tumor cells by macrophages when the inhibitory SIRP-α receptor on macrophages is blocked by antibodies from detecting its ligand, CD47, on the same tumor target cells (36). The SLAMF7 activation signaling was independent of the cytoplasmic tyrosines, but instead relied upon signaling through Mac-1 association with DAP12 and FcR-γ, as well as their operative protein tyrosine kinases, Syk and Btk (36).

## Fc-dependent NK cell activation by elotuzumab through FcγRIIIA (CD16)

Elo is an IgG1 mAb and thereby possesses an Fc domain that is capable of efficiently binding to CD16 on the surface of leukocytes. FcγRIII is expressed in two distinct forms that exhibit nearly identical extracellular amino acid sequence: (1) as a transmembrane receptor, designated FcγRIIIA, or (2) as a glycophosphatidylinositol-linked surface receptor, called FcγRIIIB (78, 79). FcγRIIIA is expressed on the surface of the cytolytic CD56^dim^ subset of NK cells, as well as intermediate and non-classical monocytes and macrophages, and can trigger potent intracellular signaling, including tyrosine phosphorylation and calcium mobilization, through physical association with the transmembrane adaptor proteins TCR-ζ and FcR-γ, which contain immunoreceptor tyrosine-based activation motifs (ITAM) (79). In contrast, FcγRIIIB is expressed on the surface of neutrophils, and signals through interactions with FcγRIIA (CD32A) (80).

Elo was found to trigger robust ADCC responses by NK cells through Fc-dependent interaction with FcγRIIIA. In the early preclinical studies by Hsi et al., HuLuc63-mediated lysis of myeloma cell lines in cultures with PBMC or in SCID mice was significantly impaired by blocking CD16 with antibodies, using an Fc mutant form of HuLuc63 with reduced CD16 binding capacity, or depleting NK cells (18). These results strongly implied that the *in vitro* and *in vivo* anti-tumor activity of Elo is mediated primarily by NK cells in a CD16-dependent manner. *In vitro* Elo begins to stimulate ADCC responses at concentration around 0.1 ng/ml, with peak responses in the range of 100 ng/ml (19, 39). However, early preclinical studies in mice found that serum concentrations that generated the most effective responses to myeloma cell lines was 70–430 μg/ml, whereas no biological activity was observed with serum concentrations of < 1 μg/ml (19).

## Alternative mechanisms of action

**Figure 2:**
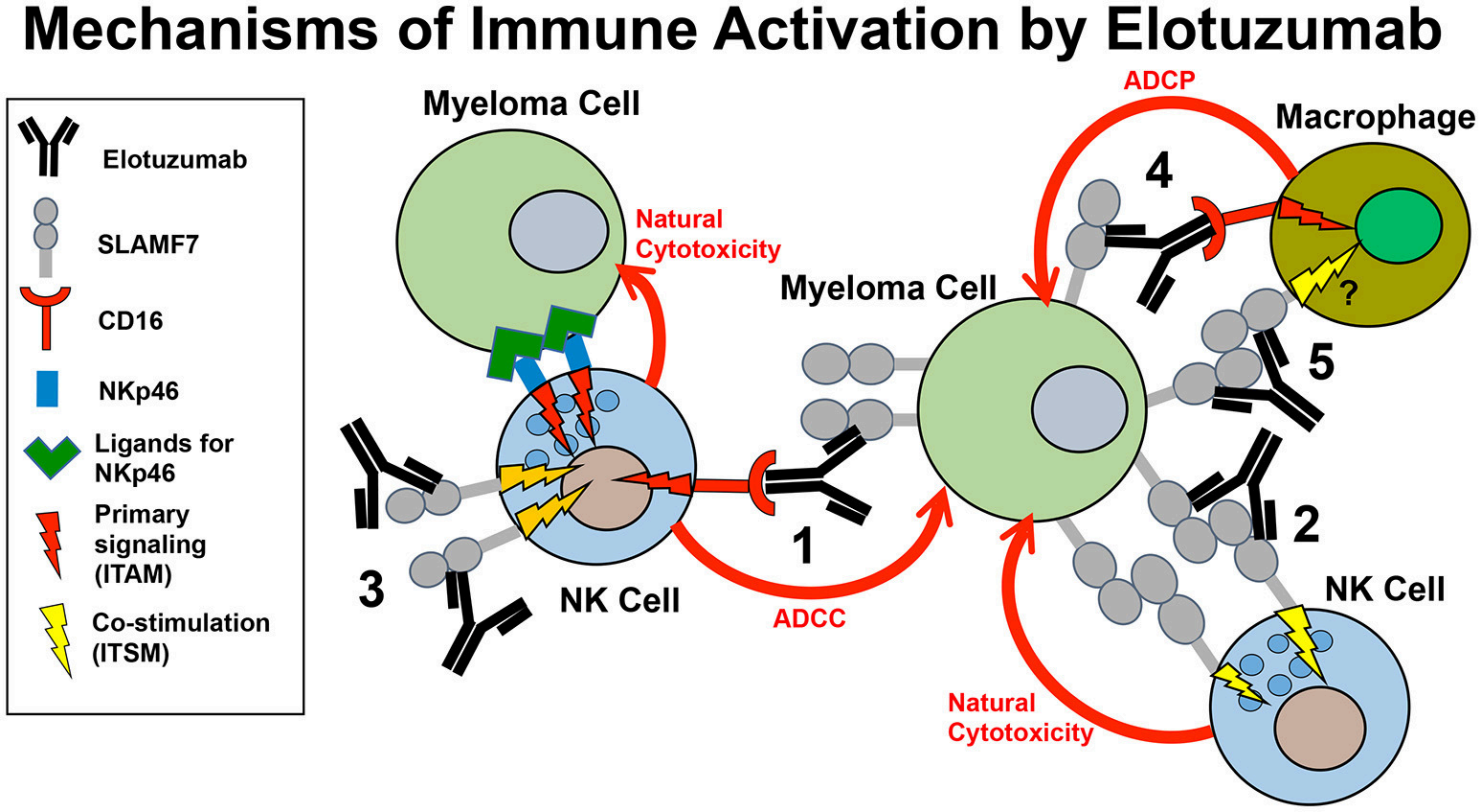
Model of the mechanisms of innate immune activation by elotuzumab. Elotuzumab promotes numerous innate immune mechanisms to enhance attack of myeloma tumor cells by NK cells and macrophages. The known mechanisms are: (1) Facilitating NK cell-mediated antibody-dependent cellular cytotoxicity (ADCC) of myeloma cells through Fc-dependent interactions with FcγRIIIA (CD16). (2) Promoting SLAMF7-SLAMF7 interactions to enhance ITSM-mediated co-stimulatory signaling in NK cells, thereby potentiating natural cytotoxicity of myeloma cells. This mechanism likely requires simultaneous engagement of ITAM-linked activating receptors on NK cells with ligands on myeloma cells. (3) Triggering ITSM-mediated co-stimulatory signaling in NK cells to enhance calcium signaling originating from ITAM-linked activating receptors (such as NKp46 or CD16) engaging with ligands on myeloma cells. (4) Promoting macrophage-mediated antibody-dependent cellular phagocytosis (ADCP) of myeloma cells through Fc-dependent interactions with Fcγ receptors. The operative Fcγ receptors in macrophages that can promote ADCP are FcγRIIIA (CD16), FcγRIIA (CD32), and FcγRI (CD64). (5) Although not yet established, it is possible that elotuzumab may also be able to promote SLAMF-SLAMF7 interactions and co-stimulatory signaling to enhance ADCP in macrophages expressing both SLAMF7 and EAT-2.

In addition to boosting ADCC responses by NK cells, several alternative immune-promoting mechanisms of action may contribute to the anti-myeloma responses of Elo in patients, as detailed in this section. These alternative mechanisms include promoting SLAMF7-SLAMF7 interactions between NK cells and myeloma cells, co-stimulating calcium signaling by other activating receptors in NK cells, and promoting ADCP of myeloma cells by macrophages (Figure 2).

### Alternative mechanisms involving NK cells

Collins et al. were the first to provide *in vitro* evidence that Elo can induce NK cell activation through direct binding to SLAMF7 on NK cells (37). The bulk of their studies used purified primary NK cells from healthy donors treated for 24 h with 100 μg/ml Elo prior to addition to assays. The addition of either Elo or F(ab')_2_ Elo to purified NK cells was found to induce expression of the activation marker CD69 in an Fc-independent manner, whereas granzyme B release (degranulation) required the Fc domain on Elo, characteristic of ADCC responses (37). In contrast, when MM target cells were added, granzyme B was released by NK cells that had been pretreated with Elo or a mutant form of Elo (G2M3) with reduced CD16-binding capacity. It should be noted that the actual mutations in Elo-G2M3 were not described and lack of affinity toward CD16 was not demonstrated (37). Nonetheless, this Fc-independent activation of NK cells suggested a mechanism involving direct engagement with SLAMF7 by the Fab domains of Elo. In accordance with these results, the CD16^−^ SLAMF7^+^ NK-92 cell line was stimulated by Elo to kill SLAMF7^+^ target cells (37). This CD16-independent cytotoxicity did not occur toward SLAMF7^−^ target cells, suggesting that Elo was “stabilizing” the SLAMF7 between NK and target cell to promote NK cell-mediated cytotoxicity. Furthermore, the effect was unique to Elo, since another SLAMF7 antibody that inhibits SLAMF7-SLAMF7 homotypic interactions instead inhibited cytotoxicity of SLAMF7^+^ target cells by NK-92 cells (37). Although further work is necessary to convincingly prove this mechanism, the evidence from Collins et al. suggest that Elo can also facilitate SLAMF7-SLAMF7 interactions between NK cells and myeloma cells to promote natural cytotoxicity, in addition to its capacity to promote ADCC responses. Although currently unpublished, Pazina et al. recently presented further evidence that Elo has unique properties among several SLAMF7 antibodies in facilitating SLAMF7-SLAMF7 interactions between NK cells and myeloma cells at the 2018 European Hematology Association meeting (81).

Pazina et al. also tested for CD16-independent effects of Elo on activation of NK cells within healthy donor peripheral blood mononuclear cells (PBMC) *in vitro* (39). Elo strongly promoted degranulation of NK cells that required integrity of the Fc domain and correlated with SLAMF7 expression level on the myeloma cells, thereby confirming that the main mechanism of action of Elo is NK cell-mediated ADCC (39). Pazina saw CD69 induction on NK in PBMC, but it was Fc-dependent, so mediated by opsonization of other SLAMF7^+^ immune cells and engaging CD16 on the NK cells. In stark contrast, no increase in CD69 expression (overnight assay) or degranulation (2 h assay) was detected on NK cells when PBMC were incubated with F(ab')_2_ Elo or an Fc mutant form of Elo that they showed to be incapable of binding to recombinant CD16 (39). Given these results, it is unclear why Collins et al. observed Fc-independent CD69 induction on purified NK cells after 24 h treatment with F(ab')2 or Fc mutant (G2M3) Elo, but perhaps due to their use of purified NK cells (39). Pazina et al. also showed that a non-fucosylated form of Elo that exhibited higher affinity toward CD16 induced more potent degranulation and CD69 expression in the presence or absence of myeloma cells, thereby providing further support for the role of the Fc domain interacting with FcγRIIIA. Whereas, Lee et al. demonstrated that the 1G2 mAb could trigger intracellular calcium mobilization upon engaging SLAMF7 expressed in a rat NK cell line (59), Pazina et al. found that crosslinking SLAMF7 with Elo alone had no impact on intracellular calcium concentrations in primary NK cells (39).

Despite defining the clear importance of the ADCC response, Pazina et al. also detected a novel co-stimulatory signaling effect that resulted when Elo engaged with SLAMF7 on the NK cell surface (39). They found that Elo binding to SLAMF7 can significantly enhance the intensity of intracellular calcium responses triggered by the ITAM-linked NK cell activating receptor, NKp46, and this co-stimulatory effect was independent of the Fc domain of Elo (39). Interestingly, while the calcium signaling required multimeric crosslinking of biotinylated NKp46 antibody with streptavidin, the Elo was not biotinylated, so was not forcibly co-aggregated with the NKp46 receptors (39). This is important, since it suggests that Elo binding to SLAMF7 on the surface of NK cells (as would occur in treated patients) can co-stimulate calcium signaling responses triggered by NKp46 and other ITAM-coupled receptors engaging with ligands on the surface of myeloma cells. In addition, non-biotinylated Elo could even further boost calcium signaling beyond levels achieved with the combination of biotinylated antibodies to NKp46 and the co-stimulatory NKG2D receptor (39). In this way, Elo demonstrates unique co-stimulatory signaling capacity, presumably resulting from SLAMF7 recruiting EAT2, which recruits PLC-γ to initiate intracellular calcium mobilization (66). It is also important that this co-stimulation effect by Elo has the potential to enhance specific tumor target cell recognition through other activating receptors, but would not universally activate NK cells in a tumor non-specific and potentially autoimmune manner. Interestingly, a similar boost in calcium signaling was previously observed in mouse NK cells stimulated with antibodies toward 2B4 + CD16, as compared to CD16 alone, and the effect was nearly lost in NK cells from EAT-2-deficient mice (66). On the other hand, biotinylated Elo plus streptavidin was unable to stimulate calcium mobilization in human NK cells by Pazina et al., whereas previous work with other SLAMF7 antibodies and secondary crosslinkers stimulated strong calcium mobilization in mouse and human NK cells (59, 61), indicating that Elo has unique properties, presumably through binding a distinct epitope within the C2 domain of SLAMF7.

### Alternative mechanisms involving other immune cells

Recently, Kurdi et al. found that Elo can also stimulate ADCP of tumor cells by tumor-associated macrophages (TAMs) in an Fcγ receptor-dependent manner (77). Elo significantly reduced tumor burden and prolonged survival in a xenograft mouse model using SCID-beige mice implanted with a myeloma cell line. SCID-beige mice lack T and B cells and have compromised NK cell cytolytic function (82), leaving monocytes/macrophages as the primary anti-tumor effector cells. The effects were abrogated using a form of Elo with the Fc domain mutated to prevent interactions with Fcγ receptors or if macrophage function was compromised by using NOD SCID gamma (NSG) immunodeficient mice (77). Elo also enhanced infiltration of TAMs, which displayed higher expression of activation markers. Finally, TAMs that had been polarized to the M1 phenotype in culture demonstrated enhanced *in vitro* ADCP capacity toward myeloma cells in the presence of Elo (77).

Although the Kurdi et al. study relies on the interactions of mouse Fcγ receptors with the humanized Elo antibody, the results open a new chapter of understanding by showing that TAMs may be an additional innate immune effector cell contributing to the mechanism of Elo anti-tumor activity in human patients (77). ADCP by monocytes and macrophages can be triggered through their surface expression of FcγRIIIA (CD16), FcγRIIA (CD32), or FcγRI (CD64) and can contribute significantly to anti-tumor effects of IgG antibodies, such as rituximab (83–86). Of note, the Fc domain of Elo has been shown to bind with approximately 5,000-fold higher affinity to CD64 than to the high affinity isoform of CD16 (39), exemplifying the potential biological relevance of this mechanism. It should be further noted that depletion of NK cells in an immunocompetent xenograft mouse model by Bezman et al. significantly reduced the anti-tumor effects of Elo, but activity was not completely lost (25). This result indicates that NK cells play a major role in Elo function, but other immune cells are also involved. Furthermore, Kurdi et al. found that either depletion of NK cells or macrophages resulted in essentially identical loss of the anti-tumor benefits of Elo in immunocompetent xenograft mice (77), although it is unclear if these results in a mouse model phenocopy the roles of these innate effector cells in humans. In addition, Bezman et al. found significantly enhanced tumor growth if CD8^+^ T cells were depleted in combination with Elo, thereby further implicating a cooperative role for cytotoxic T cells in the anti-tumor function of Elo (25). Taken together, these mouse studies demonstrate that NK cells are key effectors in mediating the biological anti-myeloma effects of Elo through ADCC and direct engagement of SLAMF7, but significant contributions are likely also derived from ADCP by TAMs, as well as supporting adaptive immune responses involving cytotoxic T cells, at least in these mouse models.

## Future research and clinical trials

In summary, accumulating published evidence demonstrates that Elo mediates strong ADCC by NK cells, enhanced SLAMF7-SLAMF7 interactions, co-stimulatory signaling in NK cells, and ADCP by macrophages (Figure 2), but additional questions remain to fully elucidate the mechanism of action by which Elo boosts immune function toward MM in patients. In addition, new preclinical studies and clinical trials are needed to develop additional effective combination therapies, to establish roles of other immune cells in Elo function, and to find biomarkers that identify patients that will best respond to Elo therapy.

A variety of mechanistic questions also remain to fully understand the mechanism of Elo activation of NK cells. For instance, accumulating data suggest that Elo binds to a unique epitope on SLAMF7 to mediate co-stimulation or facilitate SLAMF7-SLAMF7 interactions. Improved understanding of this binding site and how binding influences SLAMF7 structure, orientation, etc. are of high interest. In addition, while elegant SLAMF7 signaling function studies have been performed on NK cells in mice, particularly knockout models, more mechanistic studies in human NK cells are needed.

As with other immunotherapies, certain MM patients respond substantially better to Elo therapy, and further work is necessary to identify molecular characteristcs that are unique to high-responding vs. low-responding patients. Such findings could result in the identification of biomarkers that stratify the patients most likely to respond and tailor their therapy accordingly. For example, little is known about the expression of SLAMF7-L vs. SLAMF7-S alternative splice variants in NK cell subsets and whether these expression patterns change in subsets of MM patients or different stages of disease. Predominant expression of SLAMF7-S could render a subset of NK cells resistant to co-stimulatory signaling or perhaps inhibitory, and therefore differentially responsive to Elo. Of note, a subset of HCMV seropositive individuals exhibit “adaptive” or “memory-like” NK cell subsets lacking expression of EAT-2, and these NK cells are likely incapable of co-stimulatory signaling through SLAMF7 in HCMV seropositive individuals (68, 69) or may demonstrate inhibitory signaling through the receptor.

Since Elo is only therapeutically effective when used in combination with IMiDs or bortezomib and dexamethasone, the biological basis for these synergies require further resolution and additional combination therapies should be tested. Importantly, the mechanistic basis by which IMiDs enhance NK cell function to benefit therapeutic efficacy of Elo are largely unexplored. It was previously shown that lenalidomide can lower the threshold of NK cell activation to promote cytotoxicity and IFN-γ responses (87), but this has not yet been studied in combination with Elo. Also, early clinical trial results suggest unique synergies when pomalidomide is combined with Elo (52), raising questions of whether immune cells in addition to NK cells are contributing or if supplementation with additional immune stimulating drugs could further improve responses. Furthermore, it may be worthwhile to combine or alternate Elo therapy with the other currently available antibody that promotes NK cell-mediated ADCC in MM, daratumumab. The availability of two antibodies targeting distinct myeloma cell surface markers (SLAMF7 and CD38, respectively) provides an advantage that could be exploited in treating MM patients. In addition, expression of SLAMF7 has also been identified on tumor cells in a subset of patients with chronic lymphocytic leukemia, myelodysplastic syndrome, diffuse large B cell lymphoma, and peripheral T cell lymphoma. Therefore, other hematopoietic malignancies may be amenable to treatment with Elo.

The pre-clinical finding that non-fucosylated Elo is more potent in stimulating NK cell activation than conventional Elo (39) suggests that this enhanced form of the mAb could have improved efficacy in the clinic. While the potential toxicity of hyperactive ADCC responses by NK cells or ADCP responses by macrophages must be considered, non-fucosylated Elo may, in fact, have efficacy as a single agent or may have even better efficacy in combination therapies.

Further research is also clearly warranted to improve our minimal understanding of the impacts of Elo on anti-myeloma responses by SLAMF7-expressing monocytes, macrophages, and DC. The discoveries that SLAMF7 is highly expressed on M1 macrophages (73) and Elo promotes ADCP by inflammatory M1 macrophages (77) provide an exciting new direction that has only been minimally studied to date. Furthermore, it is interesting that non-classical “patrolling” monocytes express CD16, SLAMF7 and EAT-2 (39, 76), which are the same molecular components necessary for NK cell activation by Elo and likely contribute to ADCP and possibly co-stimulatory signaling through direct SLAMF7 engagement (Figure 2). Finally, studies are needed to test whether Elo activity is affected by SLAMF7 expression on other cells, such as subsets of B cells and CD8^+^ T cells.

Taken together, Elo has proven to be safe and effective when used in combination therapy to treat MM. Although our understanding is rapidly expanding, this unique antibody offers a wide array of additional opportunities for performing further research and for conducting new combination clinical trials to improve efficacy in treating MM and potentially other hematopoietic cancers.

## Author contributions

All authors listed have made a substantial, direct and intellectual contribution to the work, and approved it for publication.

### Conflict of interest statement

TP was supported by grants to KC and AC from Bristol-Myers Squibb (BMS), which markets elotuzumab. KC and AC have served as consultants and on scientific advisory boards for BMS.
